# SnapFib: An easy build Arduino based tabletop prototype for thin film deposition by Successive Ionic Layer Adsorption and Reaction method

**DOI:** 10.1016/j.ohx.2022.e00347

**Published:** 2022-08-19

**Authors:** Ashoke Kumar Sen Gupta, Abu Adnan, Shantanu Bhattacharjee, Nipu Kumar Das, M.A. Matin, Muhammad Quamruzzaman

**Affiliations:** Department of Electrical and Electronic Engineering, Renewable Energy Laboratory, Chittagong University of Engineering and Technology (CUET), Chattogram 4349, Bangladesh

**Keywords:** Arduino, Thin film, SILAR

## Abstract

Non-vacuum-based techniques are suitable for thin-film deposition with precision stoichiometric control. Among those, the Successive Ionic Layer Adsorption and Reaction (SILAR) method is gaining popularity for its aqueous-based almost room temperature deposition option. This method has many advantages, including the ability to control the elemental composition and stoichiometry of precursors. It is also suitable for large-area deposition. It has many runtime parameters, e.g., the number of cycles, dip time, rinse time, etc., that control the quantitative and qualitative physical properties of the deposited film. But manually controlling all these parameters for the whole process is very difficult and cumbersome. Although there are several reports published on this similar type of home-built prototype, for fast, accurate, and economically affordable deposition operations, we need to develop a machine that maintains all the properties of the SILAR process and can be made using cheap technologies. Here we report the SnapFib, a cost-effective automated tabletop prototype machine that is easy to build for thin-film deposition on soda-lime glass substrates by the SILAR method without almost any human intervention. SnapFib is built using linear actuators, an ATmega328P (a microcontroller available on Arduino boards), and some other parts collected from laboratory sites. The whole firmware needed for this device has been developed and maintained using the Arduino IDE (Integrated Development Environment). All required functional features and control parameters are encoded in the microcontroller firmware. The construction cost of this prototype is around 600 USD. We validated our construction through XRD (X-ray Diffraction) and FESEM (Field Emission Scanning Electron Microscope) characterizations of thin films that were deposited by SnapFib. Since this is built under the CC-BY license, students and researchers can freely perform and validate their experiments and modify the hardware and software as required. With how easy it is to make and how much it costs; we hope that many thin-film deposition labs will quickly start using SnapFib as an added benefit.

## Graphical abstract

**Figure:**
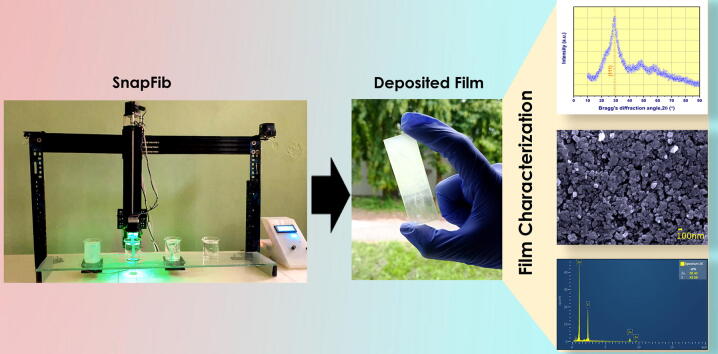

**Specifications table:** 

Hardware name	SnabFib – An open-source Arduino-based thin film deposition system
Subject area	Engineering and materials science
Hardware type	Material synthesis using the SILAR method
Open-source license	CC BY 4.0
Cost of hardware	$600 USD
Source file repository	https://doi.org/10.17632/3pccd4yp8m.4

## Hardware in context

Thin films of materials are deposited on flexible or flat substrates (e.g., soda-lime glass) mainly by two techniques: vacuum and non-vacuum. Common vacuum-based techniques are sputtering, thermal evaporation, close-spaced sublimation, pulsed laser deposition, etc. Most of them are very expensive and require a lot of space to be accommodated with other machinery (e.g., vacuum pumps) to run the system. As a result, those machines cannot be built in a home environment from scratch. On the other hand, machines that use non-vacuum-based techniques are comparatively cheaper, unless otherwise smart features are added; they are easy to operate, and possible home-built prototype options are available. Most non-vacuum-based techniques use solid solution chemical synthesis routes, namely spin coating, doctor blading, dip coating, SILAR, etc. Both thin-film deposition processes have their own set of merits and demerits. As non-vacuum-based systems use chemical synthesis routes, controlling elemental composition ratios and atomic percentages in the final fabricated film takes it upfront among the researchers’ community.

To grow thin films with the SILAR method, the substrate is successively immersed into separate cationic and anionic precursor solutions. It is found that at least four deposition steps are required to complete a SILAR cycle, in general. Adsorption in the cationic precursor solution, rinsing in deionized water, the reaction in the anionic precursor solution, and rinsing in deionized water are the steps. The underlying physical and chemical principles of the SILAR method are reported in the article [1]. The SILAR method is popular as it uses aqueous media as a solvent for precursor salts as compared to other toxic aprotic solvents like 2-Methoxyethanol. With some exceptions, SILAR depositions are performed mainly at room temperature. For thin film deposition recipes that need a temperature above room temperature but below the boiling point of water, extra features like a temperature controller option must be added to the system at an extra cost.

SILAR systems that are commercial quality and have complete functionality and smart features are very expensive. When the goal is to deposit a single layer film of a few micrometers in a device made up of multiple layers of films, such as a thin-film solar cell, purchasing a whole piece of commercial-grade SILAR equipment is not justified. This needs thin-film deposition methods based on the SILAR approach in home-built devices [1], [2], [3], [4]. Numerous designs and structures for automating the deposition process have been proposed [5], [6]. Though some devices have been implemented, they are quite complex and expensive. SnapFib is a tabletop open-source thin-film deposition machine that we demonstrate in this article. Experiments were conducted to validate the prototype SnapFib thin-film fabrication. In this article, we present SnapFib: an open-source automated tabletop machine for thin film deposition controlled by an Arduino-based system. We also report validating experiments that demonstrate the capabilities of this low-cost and easy-to-build prototype for thin film fabrication.

## Hardware description

**Fig. 1:**
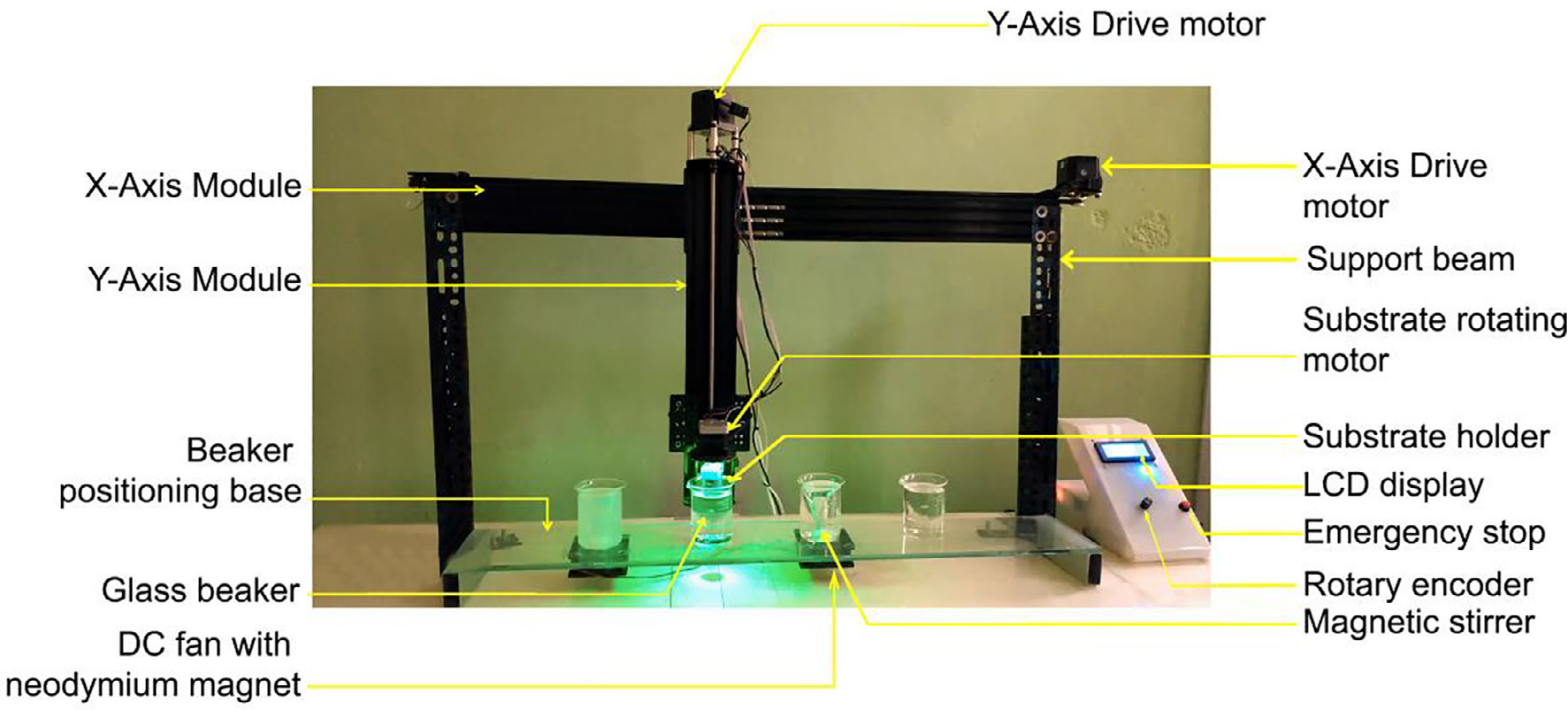
An overview of the designed hardware.

SnapFib’s closed-loop control mechanism assures that the 2-axis operation is vibration-free, smooth, and practically silent. To assure dust-free functioning, the entire SnapFib is housed in a box made of clear acrylic boards. Our SnapFib prototype is also complete in that it is affordable, uses minimal hardware that is easy to change, doesn't have licensing restrictions on user changes, and gives users the freedom to add more features. An overview of the designed hardware is shown in Fig. 1.

The presented hardware also has the following additional features:

• Vertical and horizontal movement is achieved using a combination of V-slot lead screw and belt-pulley powered linear actuators. Actuators with lead screws are expensive. Both types of actuators may be used for movement, but in our designed hardware, they will significantly reduce the budget.
• Using a rotary encoder instead of a normal keypad to choose and set control settings makes the process quick and easy.
• A Nema17 stepper motor and a 3D-printed substrate holder are used to make the rinsing move.
• The magnetic stirring option may be controlled.
• Using an emergency stop switch makes SnapFib safer in case there is a mechanical or electrical problem.
• Electronically controlled by an Arduino-based system (due to its good documentation and support, as well as its very simple Integrated Development Environment (IDE), Arduino has recently been adopted by other researchers to control their ad-hoc thin film fabrication systems [5], [7], [8], [9].
• One important change in the future could be to do thin-film deposition in an atmosphere of inert gas (N_2_ or Ar).

The cost of two commercially available SILAR systems is given below:

1. SILAR Coating System with Magnetic Stirrer (Model: HO-TH-03B) by Holmarc: $ 7000 USD [10].

2. SILAR Coating System with Magnetic Stirrer (Model: HO-TH-03B1) by Holmarc: $ 7000 USD [11].

Compared to the commercial systems, our system costs only $ 600 USD which is about 90 % cheaper.

The key differences between our proposed system with the commercially available systems are:

1. For the proposed system our focus is on using minimal hardware, automation with minimal human intervention, user-friendliness, and almost vibration-free operation with quality thin-film deposition.
2. To adopt minimal hardware, we have discarded the aesthetic beauty of hardware, inert environment for the deposition, temperature controller features, etc. which are the most common features of a commercially built system.
3. The system is suitable for a single thin-film or one/two of the thin-film layers in a stack of cascaded layered devices (e.g., in a complete thin-film solar cell device).
4. As a result, the designed system offers low-cost build, affordability, and adaptability among the researchers.

## Hardware description in details

To describe SnapFib hardware in its simplest form, we can divide it into four main modules in a structure that goes from the bottom up above the table base.

**Fig. 2:**
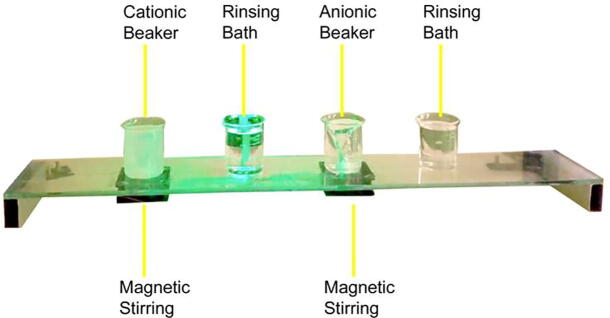
Beaker positioning base with magnetic stirrer features.

1. Beaker positioning base with magnetic stirring features (Beaker positioning module)
2. Belt-pulley driven linear actuator system (X-axis module)
3. Lead-screw linear actuator system (Y-axis module)
4. Power supply and control unit (Electronic module)

• Beaker positioning module:

This part is designed to hold four 150 mL glass beakers in four evenly spaced places, 1-2-3-4. The dipping glass substrate on which the location of the beaker may be read from the LCD (Liquid Crystal Display) while the machine is running. A SILAR cycle is considered to be completed when the glass substrate passes through four locations. In addition to the standard 1-2-3-4 (adsorption-rinsing-reaction-rinsing) movements, other combinations (e.g., 1122, 1221, 4321, etc.) can be programmed as needed for specific investigations. Fig. 2 shows the designed beaker positioning module along with the magnetic stirrers. Magnetic stirring of liquids in the beaker is possible in two of the four positions. This is done by cutting a hole in the acrylic that is smaller in diameter than the beaker and putting a DC fan underneath it. Two neodymium magnets are attached to the DC fan, in opposite pole faces with respect to one another. We used Teflon coated magnetic stirrer bar inside the beakers containing solutions. Those bars rotate as an interaction with the magnetic fields created by the magnets upon the rotation of the DC fan. Of course, stirring actions can be programmed depending upon the deposition conditions from the control panel.

**Fig. 3:**
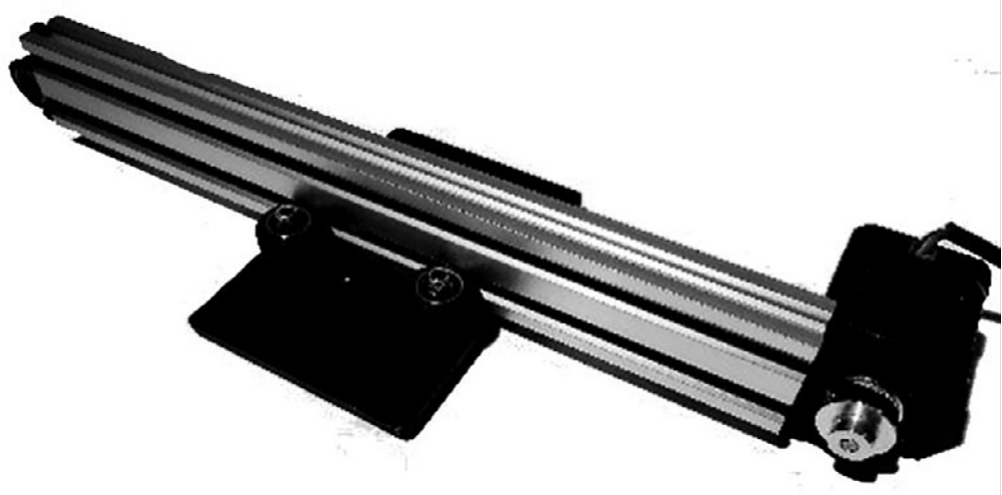
Belt-pulley driven linear actuator system (courtesy: OPENBUILDS).

• X-axis module:

The X-axis module shown in Fig. 3 handles the horizontal motion of the device. A gantry plate-1 is attached on wheels with two series connected to each 500 mm long horizontal movement rail. This gantry plate is powered by a Nema23 stepper motor and is moved by a belt. It can move horizontally within two limit switches located at each end of a 1000 mm rail.

**Fig. 4:**
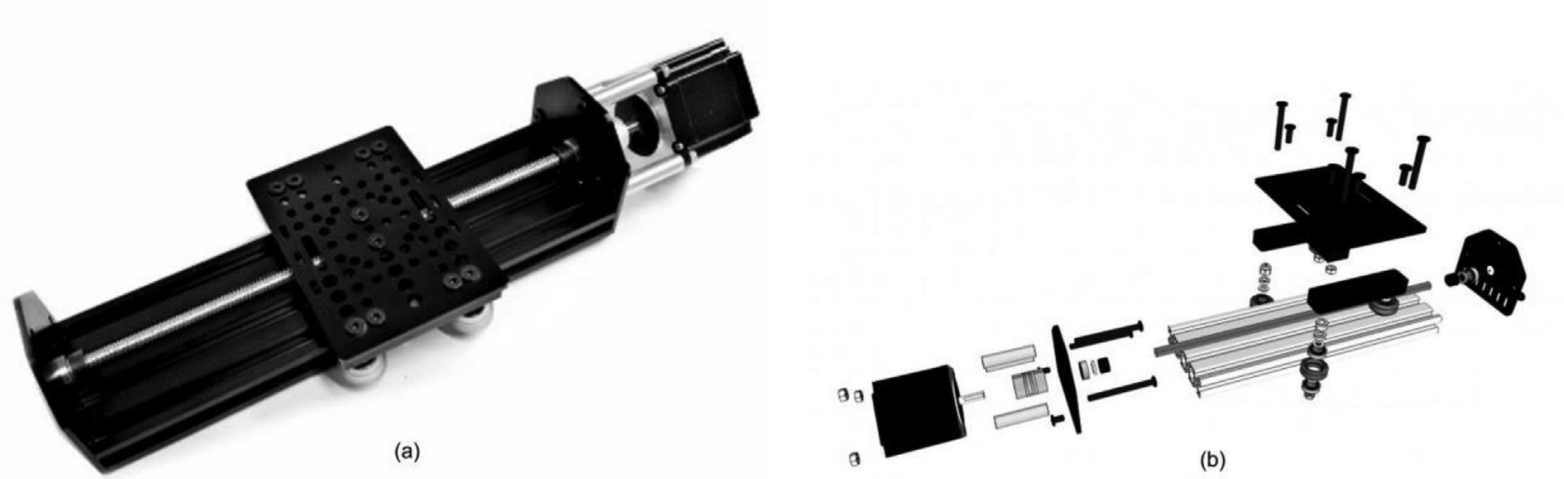
a) Lead-screw drive linear actuator module. b) 3D modeling file. (Courtesy: OPENBUILDS).

• Y-axis module:

A gantry plate-2 is attached to a V-slot lead-screw driven rail and travels up and down with a NEMA23 stepper motor rotating the lead-screw rod. There are also two limit switches to prevent it from moving vertically by mistake. The gantry plate-2 is linked with a 3D printed substrate holder and a Nema17 stepper motor. RGB LEDs are also used to embellish the substrate holder, which lights up when the system is running. By installing it on the gantry plate-1, the entire module may be moved horizontally. The total structure of the y-axis module and the 3D building blocks are shown in Fig. 4.

**Fig. 5:**
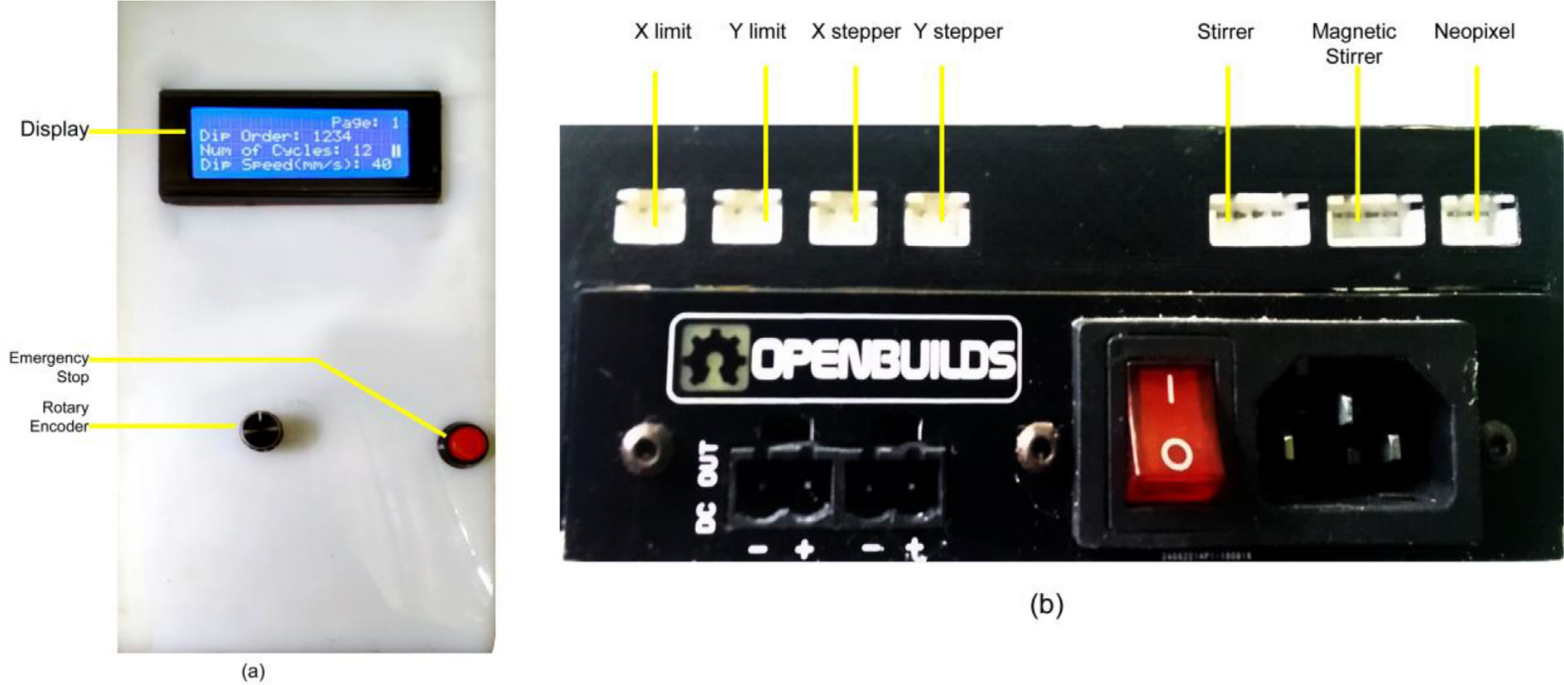
a) Control unit b) Power supply assembly.

• Electronic module:

An 8-bit microcontroller ATmega328P-based control panel is designed to control the parameters of the SILAR depositor. We can easily control several parameters, like the number of cycles, dip time, dip speed, dip length, etc. There are two beakers that are equipped with magnetic stirring of the chemical solution. A magnetic stirrer can be controlled with this control panel. These magnetic stirrers are built using BLDC motors and Neodymium magnets. To show the running parameters and output status a Liquid Crystal Display has been used. This is a parallel LCD that gives us a simple and cheaper solution for using an RGB Liquid Crystal display in our system. This display provides a 20-characters by 4-line interface with clear text on blue background light. To reduce the number of I/O ports by transforming the parallel lines of the LCD to I2C compatible lines we used an I2C module that is also ROHS-compliant. The heart of the board is a PCF8474 I2C chip that is capable of transforming I2C (Inter-Integrated Circuit) serial data to parallel data suitable for the LCD. The I2C address used for the LCD in our system was found using an I2C search program and this address can be changed using three soldered jumpers on the board. This setting enables us to use up to three LCDs controlled using only one I2C bus where each has its own address. Fig. 5 shows the complete control unit and the power supply assembly.

**Fig. 6:**
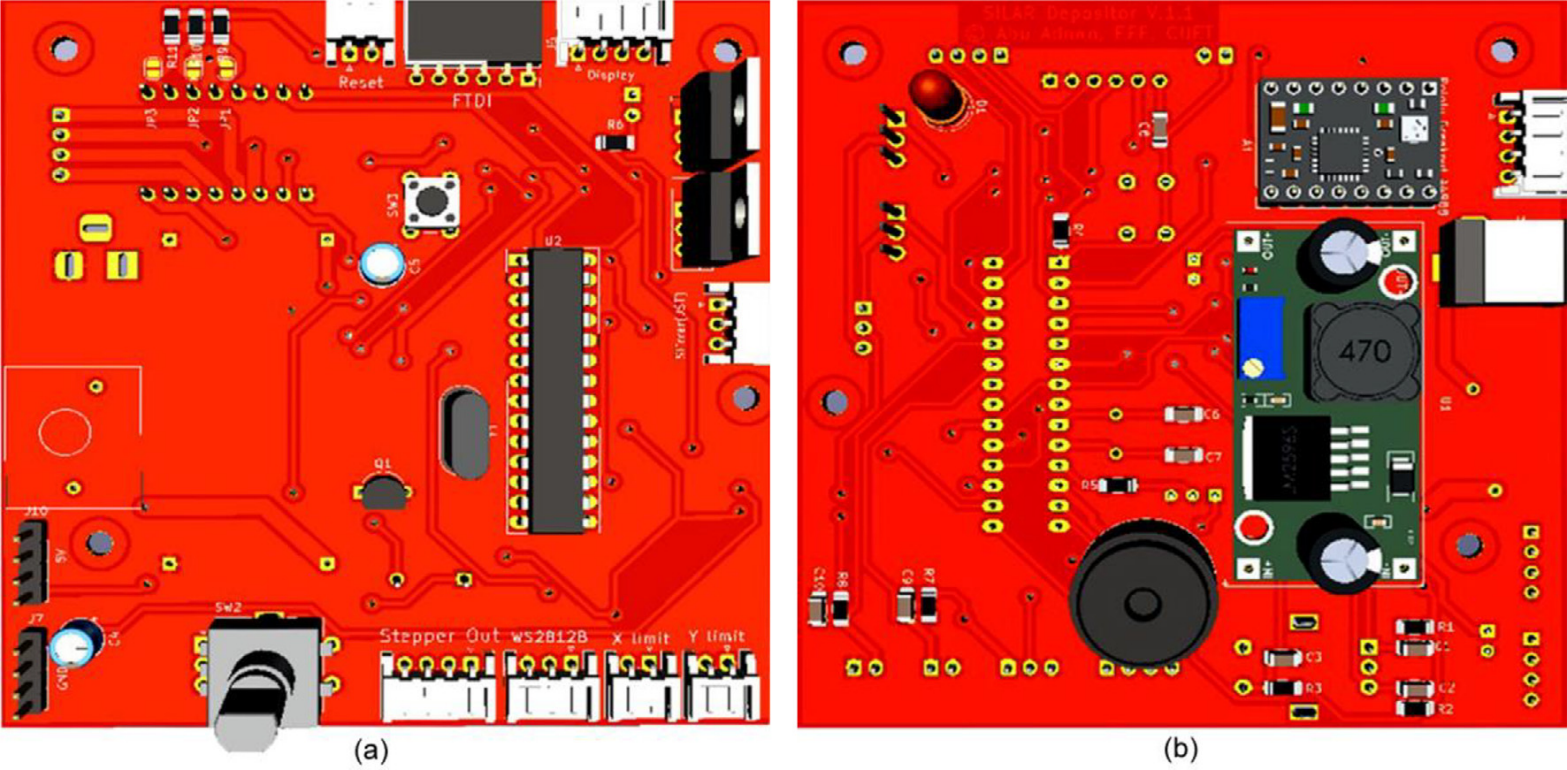
a) PCB layout of the electronic module (front part) b) PCB layout of the electronic module (back part).

There are output ports for stepper drivers, limit switches, and magnetic stirrers on the back side of the control panel shown. The electrical system architecture and connection details of the components are shown in Fig. 6.

**Table:** 

**SnabFib Parameter**	**Details**
SnabFib Dimensions	1000 mm × 500 mm × 500 mm
Max Movement Speed (X-axis, mm/s)	40 mm/s
Max Movement Speed (X-axis, mm/s)	40 mm/s
Power Input	220 V, 50 Hz, 400 Watts
**Safety Issues**	
• Do not manually move the X-axis and the Y-axis drive while in operation • Check limit switches before running. • Press the emergency stop switch for any malfunctions.

## Design files summary

**Table:** 

**Design file name**	**File type**	**Open source license**	**Location of the file**
Stepper_holder.f3d	CAD	CC BY 4.0	https://doi.org/10.17632/3pccd4yp8m.4
Silde_holder.f3d	CAD	CC BY 4.0	https://doi.org/10.17632/3pccd4yp8m.4
SILAR.kicad_pcb	PCB	CC BY 4.0	https://doi.org/10.17632/3pccd4yp8m.4
SILAR.pro	PCB	CC BY 4.0	https://doi.org/10.17632/3pccd4yp8m.4
SILAR.sch	PCB	CC BY 4.0	https://doi.org/10.17632/3pccd4yp8m.4
/gerbers	Gerber	CC BY 4.0	https://doi.org/10.17632/3pccd4yp8m.4
/arduino_firmware	Arduino sketch	CC BY 4.0	https://doi.org/10.17632/3pccd4yp8m.4
SnapFib operation	Video	CC BY 4.0	https://doi.org/10.17632/3pccd4yp8m.4
X-axis assembly	Video	CC BY 4.0	https://doi.org/10.17632/3pccd4yp8m.4
Y-axis assembly	Video	CC BY 4.0	https://doi.org/10.17632/3pccd4yp8m.4

## Bill of materials

**Table:** 

**Designator**	**Component**	**Number**	**Cost per unit -USD**	**Total cost -USD**	**Source of materials**	**Material type**
Beaker positioning module	Aluminum Square Tube	02	0.57	1.14	Local store	Metal
	Tempered Glass (8 mm) (6″ X 36″)	01	9.11	9.11	Local store	Other
	BLDC Fan	02	0.80	1.60	Local store	Other
	Neodymium Magnet	04	3.42	13.68	Local store	Other
	Magnetic Stirrer Bar (10 pcs/pack)	01	9.91	9.91	Local store	Other
X-axis module	V-Slot NEMA 23 Linear Actuator (Belt Driven)	01	96.99	96.99	Open Builds*	Metal
	Low Profile Screws M5 – 8 mm (10 pack)	01	0.99	0.99	Open Builds	Metal
	Aluminum Spacer – 6 mm (10 pack)	01	3.39	3.39	Open Builds	Metal
	Aluminum Spacer – 3 mm (10 pack)	01	2.49	2.49	Open Builds	Metal
	Double Tee Nuts	03	0.69	2.07	Open Builds	Metal
	GT3 (2 mm) Timing Pulley – 20 Tooth	01	5.99	5.99	Open Builds	Polymer
	Eccentric Spacers – 6 mm	02	1.99	3.98	Open Builds	Metal
	Solid V Wheel Kit	04	5.19	20.76	Open Builds	Composite
	Smooth Idler Pulley Kit	01	5.99	5.99	Open Builds	Polymer
	Idler Pulley Plate	01	6.99	6.99	Open Builds	Metal
	Motor Mount Plate for NEMA 23	01	7.99	7.99	Open Builds	Metal
	Cable Ties (10 pack)	01	0.79	0.79	Open Builds	Polymer
	Low Profile Screws M5 – 15 mm (10 pack)	01	1.19	1.19	Open Builds	Metal
	V-Slot Gantry Plate (20 mm)	01	6.29	6.29	Open Builds	Metal
	3GT (GT2-3 M) Timing Belt - By the Foot	01	3.49	3.49	Open Builds	Polymer
	V-Slot® Linear Rail – 20x40	01	3.99	3.99	Open Builds	Metal
	Nylon Insert Hex Locknut - M5 (10 pack)	01	0.99	0.99	Open Builds	Metal
	Low Profile Screws M5 – 25 mm (10 pack)	01	1.39	1.39	Open Builds	Metal
	Allen Wrench Set (1.5 mm, 2 mm, 2.5 mm, 3 mm)	01	1.56	1.56	Open Builds	Metal
	NEMA 23 Stepper Motor	01	27.99	27.99	Open Builds	Metal
Y-axis module	Nylon Insert Hex Locknuts - M5 (10 pack)	01	0.99	0.99	Open Builds	Metal
	Aluminum Spacer – 6 mm (10 pack)	01	3.39	3.39	Open Builds	Metal
	Aluminum Spacer – 40 mm (10 pack)	01	4.89	4.89	Open Builds	Metal
	Aluminum Spacer – 3 mm (10 pack)	01	2.49	2.49	Open Builds	Metal
	Low Profile Screws M5 – 40 mm (10 pack)	01	1.69	1.69	Open Builds	Metal
	Low Profile Screws M5 – 55 mm (10 pack)	01	1.99	1.99	Open Builds	Metal
	Low Profile Screws M5 – 15 mm (10 pack)	01	1.19	1.19	Open Builds	Metal
	1/4″ x 8 mm Flexible Coupling	01	6.99	6.99	Open Builds	Metal
	Eccentric Spacer – 6 mm	02	1.99	3.98	Open Builds	Metal
	Xtreme Solid V Wheel Kit	04	6.99	27.96	Open Builds	Other
	Spacer Blocks	02	5.99	11.98	Open Builds	Metal
	V-Slot Gantry Plate (Universal)	01	11.99	11.99	Open Builds	Metal
	Self-Tapping Screw	04	0.29	1.16	Open Builds	Metal
	Ball Bearing 688Z 8x16x5	02	0.99	1.98	Open Builds	Metal
	8 mm Shim	02	0.22	0.44	Open Builds	Metal
	8 mm Lock Collar	02	1.19	2.38	Open Builds	Metal
	Anti-Backlash Nut Block	01	9.99	9.99	Open Builds	Metal
	8 mm Metric Acme Lead Screw	01	10.99	10.99	Open Builds	Metal
	NEMA 23 Stepper Motor	02	27.99	55.98	Open Builds	Metal
	Threaded Rod Plate (NEMA 23)	02	8.99	17.98	Open Builds	Metal
	V-Slot Linear Rail 20x80mm	01	12.99	12.99	Open Builds	Metal
	Allen Wrench Set (1.5 mm, 2 mm, 2.5 mm, 3 mm)	01	1.56	1.56	Open Builds	Metal
Electronic module	Meanwell Power Supply Bundle (24 V)	01	69.99	69.99	Open Builds	Metal
	Power Cable	01	4.00	4.00	Open Builds	Composite
	Rotary encoder	01	2.72	2.72	Local Store	Metal
	ATmega328P	01	3.00	3.00	Local Store	Other
	Resistor (10 k)	10	0.075	0.75	Local Store	Other
	Resistor (2 2 0)	01	0.075	0.075	Local Store	Other
	Capacitor (22pF)	02	0.075	0.150	Local Store	Other
	Capacitor (100nF)	06	0.075	0.45	Local Store	Other
	Capacitor (1uF)	01	0.05	0.05	Local Store	Other
	Capacitor (10uF)	01	0.05	0.05	Local Store	Other
	LCD module	01	5.00	5.00	Local Store	Other
	NEMA 23 Stepper Motor	01	27.99	27.99	Open Builds	Metal
	DQ542MA Stepper Motor Driver	02	39.99	79.98	Open Builds	Other
	A4988 Stepper driver	01	2.25	2.25	Local Store	Other
	Buck module	01	1.00	1.00	Local Store	Other
	DC socket	01	0.125	0.125	Local Store	Other
	AMS 1117 5.0	01	0.125	0.125	Local Store	Other
	I2C display driver	01	1.00	1.00	Local Store	Other
	WS 2812B neo pixel ring	01	2.50	2.50	Local Store	Other
	IRLZ44N *N*-channel MOSFET	02	0.25	0.25	Local Store	Other
	Buzzer	01	0.125	0.125	Local Store	Other
	Micro limit switch	02	0.50	0.50	Local Store	Other
	BC547	01	0.025	0.025	Local Store	Other
	Power switch	01	0.125	0.125	Local Store	Other
	Crystal (16 MHz)	01	0.20	0.20	Local Store	Metal
	PCB	01	5.00	50	Local Store	Polymer
	JST connector (4 pins)	02	0.25	0.50	Local Store	Other
	JST connector (2 pins)	03	0.25	0.75	Local Store	Other
	JST connector (3 pins)	02	0.25	0.50	Local Store	Other
	Male header	01	0.25	0.25	Local Store	Other
	Female header (l-shaped)	01	0.25	0.25	Local Store	Other

*OPENBUILDS part store, 40,343 air time avenue, Zephyrhills, Florida 33542, USA is an open-source hardware store that provides all kinds of hardware related to CNC, 3D printers, and laser engraving.

Website: https://openbuildspartstore.com/.

## Build instructions

Parts required for building 04 modules of the entire hardware uploaded to the repository (visual). The following are the visual build instructions for each module.

Beaker positioning module:

1. Attach the two metal square tubes to the two ends of the glass in the way shown in Fig. 7 so that the tubes can handle the weight of the glass and beakers appropriately.

**Fig. 7:**
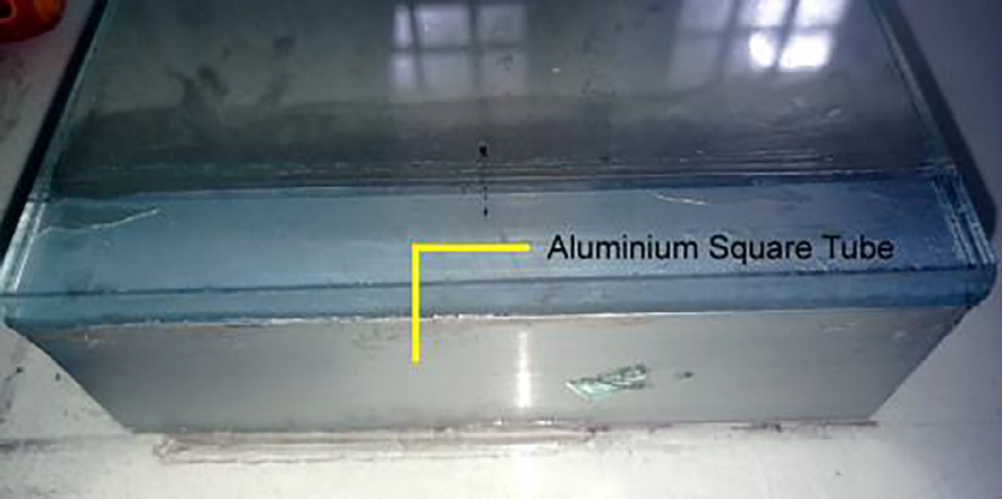
Square tube attached with a glass sheet.
2. Mark four positions for the four beakers in the tampered glass. The positions of the beakers will be in the following order from the left side of the glass: i) Beaker for cationic bath ii) Beaker for rinsing iii) Beaker for anionic bath iv) Beaker for rinsing
3. Attach two magnets on two wings of each BLDC (Brushless Direct Current) motor with proper glue. The setup is shown in Fig. 8. Make two holes in the cationic bath position and the anionic bath position such that the diameter of the holes is much smaller than the diameter of the beakers. The holes help increase the magnetic field strength for stirring.

**Fig. 8:**
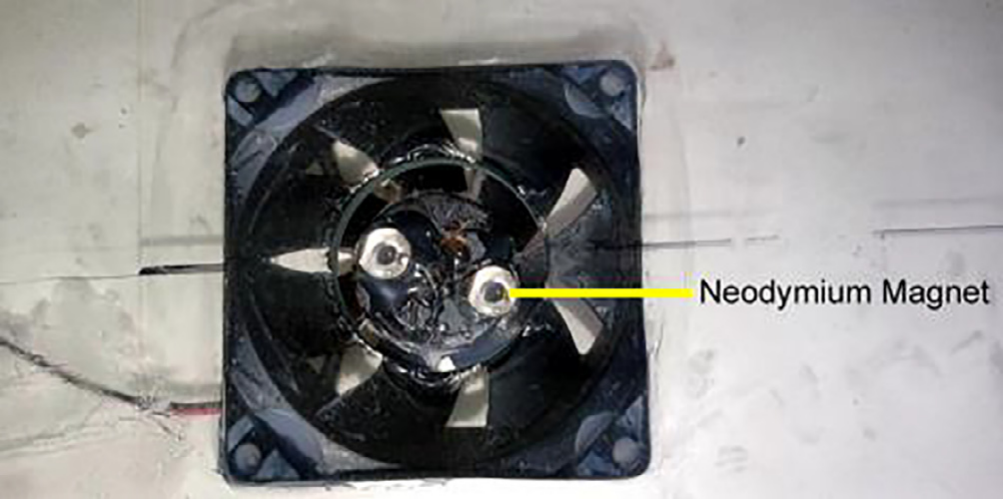
Neodymium magnet attached with BLDC fan.
4. In the positions of the cationic bath beaker and anionic beaker, attach the BLDC fan and magnet assembly on the bottom side of the glass.
5. Connect the BLDC fans to the electrical system and control unit as necessary. After necessary adjustments, the final view of the beaker positioning base is shown in Fig. 9.

**Fig. 9:**
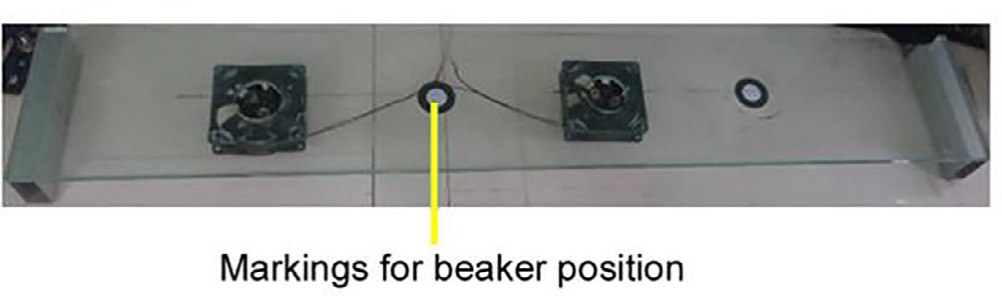
Marking for the rinsing position in the glass base.

**Fig. 10:**
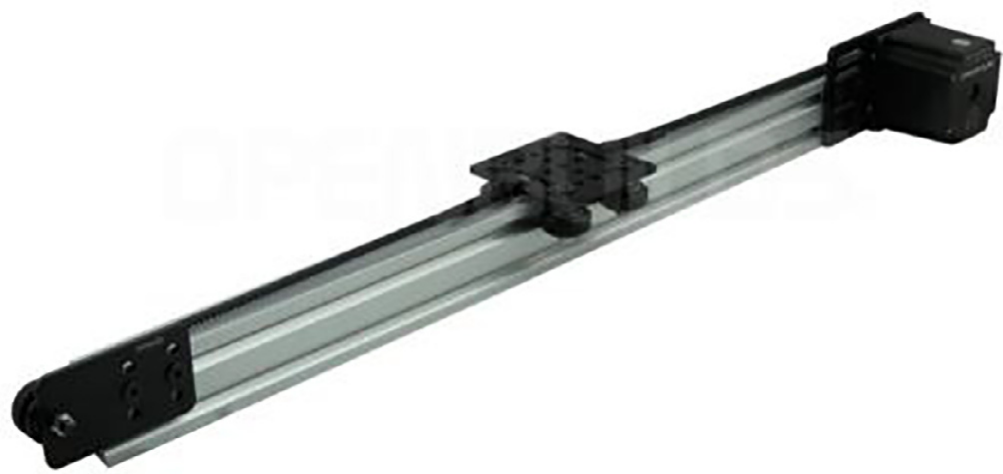
Completed Belt-pulley driven linear actuator module (Courtesy: OPENBUILDS).

**Fig. 11:**
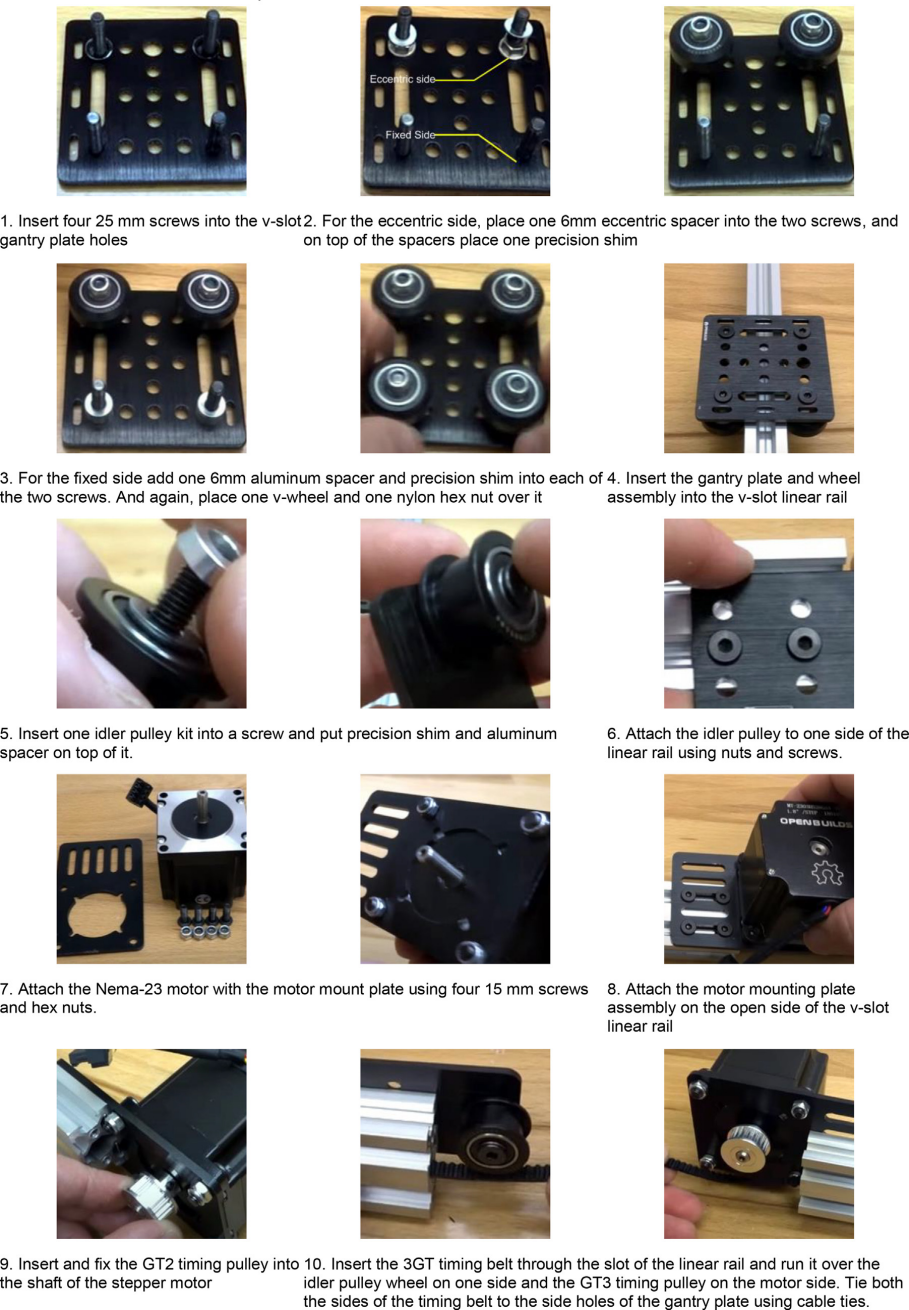
Visual guide for assembling belt-pulley driven linear actuator system (Courtesy: OPENBUILDS).

X-axis module:

1. Insert four 25 mm screws into the V-slot gantry plate holes and place the plate on its back.
2. Among the four screws, two are used for the eccentric side and the other two are used for the fixed side. For the eccentric side, place one 6 mm eccentric spacer into the two screws, and on top of the spacers, place one precision shim. After that, add one solid v-wheel and one nylon hex nut over it on each of the wholes of the eccentric side.
3. For the fixed side, add one 6 mm aluminum spacer and a precision shim to each of the two screws. Place one v-wheel and one nylon hex nut over it in each of the two holes. Thus, the gantry plate and wheelset are ready.
4. Insert the gantry plate and wheel assembly into the V-slot linear rail and adjust the spacers and hex nuts as necessary to get the proper amount of friction and movement in the wheels to be able to move along the linear rail properly.
5. Insert one idler pulley kit into a screw and put a precision shim and an aluminum spacer on top of it. Then fix the idler pulley kit with the idler pulley plate and adjust the screw as necessary.
6. Attach the idler pulley assembly to one side of the linear rail using a double tee-nut and screws.
7. Attach the Nema-23 motor to the motor mount plate using four 15-mm screws and hex nuts.
8. Use double tee-nuts and screws to connect the motor mounting plate assembly to the open side of the V-slot linear rail.
9. Insert and fix the GT2 timing pulley into the shaft of the stepper motor.
10. Insert the 3GT timing belt through the slot of the linear rail and run it over the idler pulley wheel on one side and the GT3 timing pulley on the motor side. Use cable ties to tie both the sides of the timing belt to the side holes of the gantry plate. Adjust the belt and the cable tie to ensure that the belt is neither too tight nor too loose to be able to move the gantry plate smoothly along the v-slot linear rail.
11. With some final adjustments, finish building the X-axis assembly (Fig. 10). Fig. 11 shows a step-by-step visual guide for building the X-axis assembly.

**Fig. 12:**
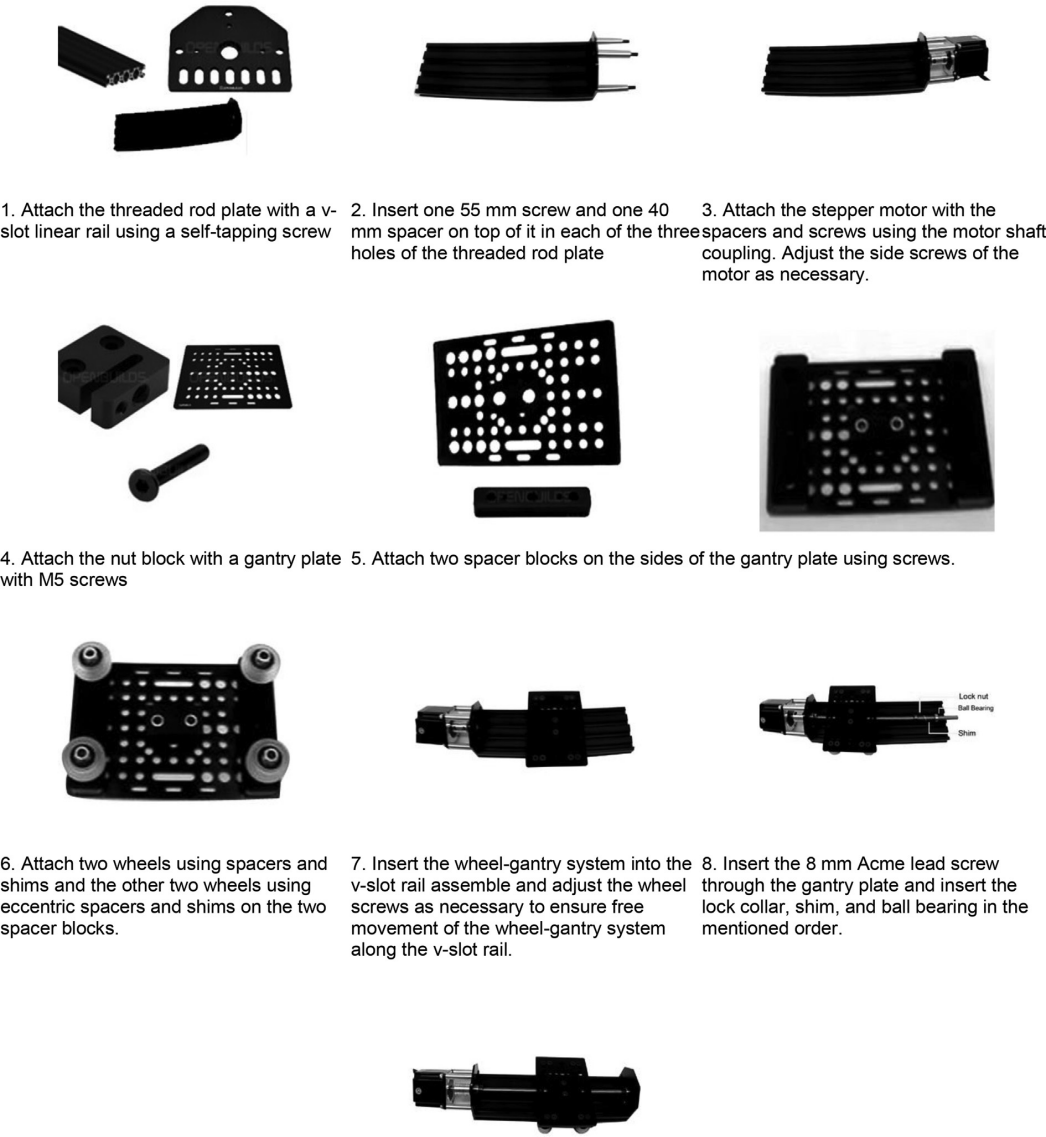
Visual guide for assembling lead-screw linear actuator system (Courtesy: OPENBUILDS).

Visual steps (X-axis assembly) as outlined above:

Y-axis module:

1. Attach the threaded rod plate to the v-slot linear rail using a self-tapping screw.
2. Insert one 55-mm screw and one 40-mm spacer on top of it in each of the three holes of the threaded rod plate.
3. Attach the stepper motor with the spacers and screws using the motor shaft coupling. Adjust the side screws of the motor as necessary.
4. Attach the nut block to the gantry plate with M5 screws.
5. Attach two spacer blocks on the sides of the gantry plate using screws.
6. Attach two wheels using spacers and shims and the other two wheels using eccentric spacers and shims on the two-spacer blocks.
7. Insert the wheel-gantry system into the V-slot rail assembly and adjust the wheel screws as necessary to ensure free movement of the wheel-gantry system along the V-slot rail.
8. Insert the 8 mm Acme lead screw through the gantry plate and insert the lock collar, shim, and ball bearing in the mentioned order.
9. Attach the remaining threaded rod plate to the end side of the V-slot linear rail and fix the lead screw to the rod plate using the lock collar shim and ball bearing. Adjust the lock collar as necessary so that the rotation of the lead screw properly moves the gantry plate up along the lead screw. The final view of the assembled Y-axis module is shown in Fig. 4(a). Fig. 12 shows a step-by-step visual guide for building the Y-axis module.

Visual steps (Y-axis assembly) as outlined above:

### Substrate holder sub-assembly with Y-axis module

1. Obtain any well-functioning 3D printer of at least 120 × 70 × 100 mm.
2. Obtain PLA filament of about 60 g minimum.
3. Change any dimension of the “Stepper_holder.f3d” and “Slide_holder.f3d” files if necessary or use the provided STL files for printing. The 3D illustration of the stepper holder and slide holder is shown in Fig. 13.

**Fig. 13:**
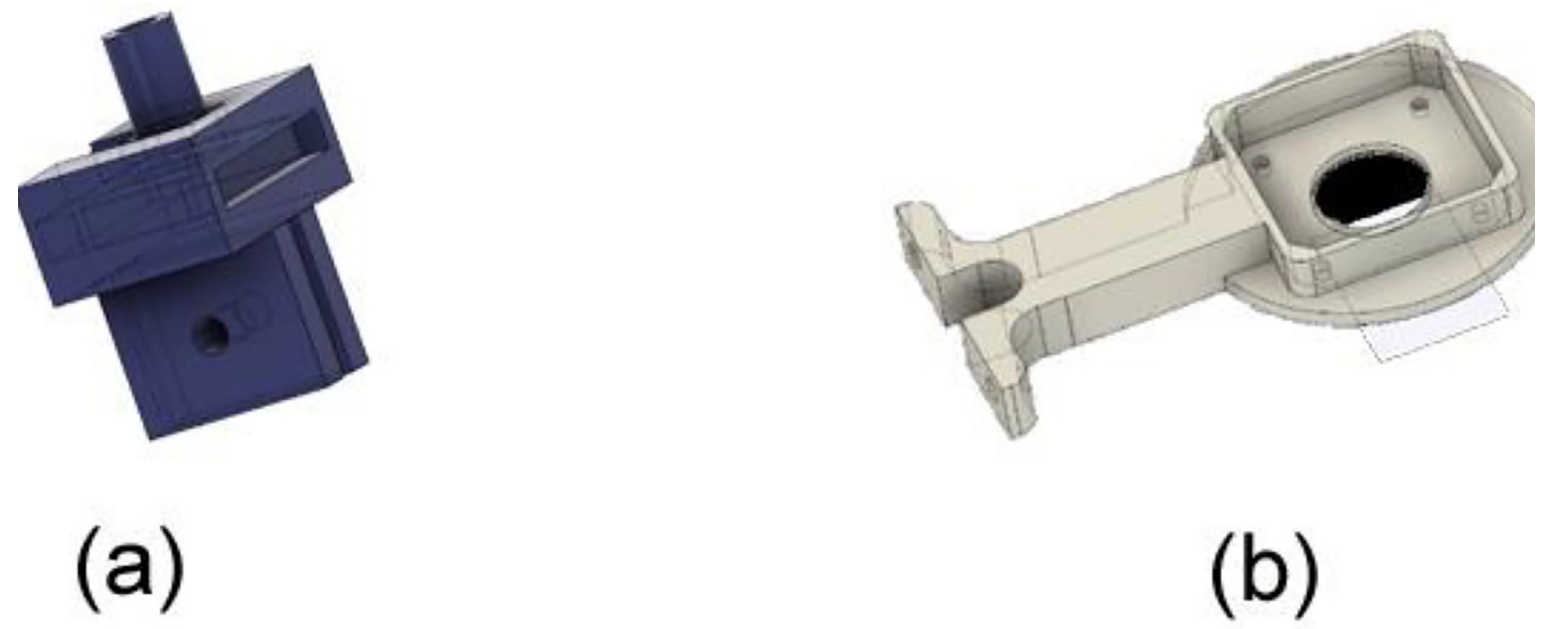
a) 3D illustration of Stepper holder b) 3D illustration of Slide holder.
4. Open the STL files using any 3D slicing software like “Cura” and change the orientation of the structures if necessary. Print the stepper holder and slide holder using the design files. Recommended settings for the printing are given below:
Layer height: 0.2 mm
Top/Bottom Thickness: 1.2 mm
Wall Thickness: 1.2 mm
Infill density: 100 % (Minimum of 70 %)
1. Print time and weight of print material are given below:
i. For “Stepper_holder” PLA material of approximately 35 – 40 g is used with a printing time of about 4 h.
ii. For “Slide_holder” PLA material of approximately 25 – 30 g is used with a printing time of about 2–3 h.
2. After the complete building process, the stepper holder and slide holder are shown in Fig. 14.

**Fig. 14:**
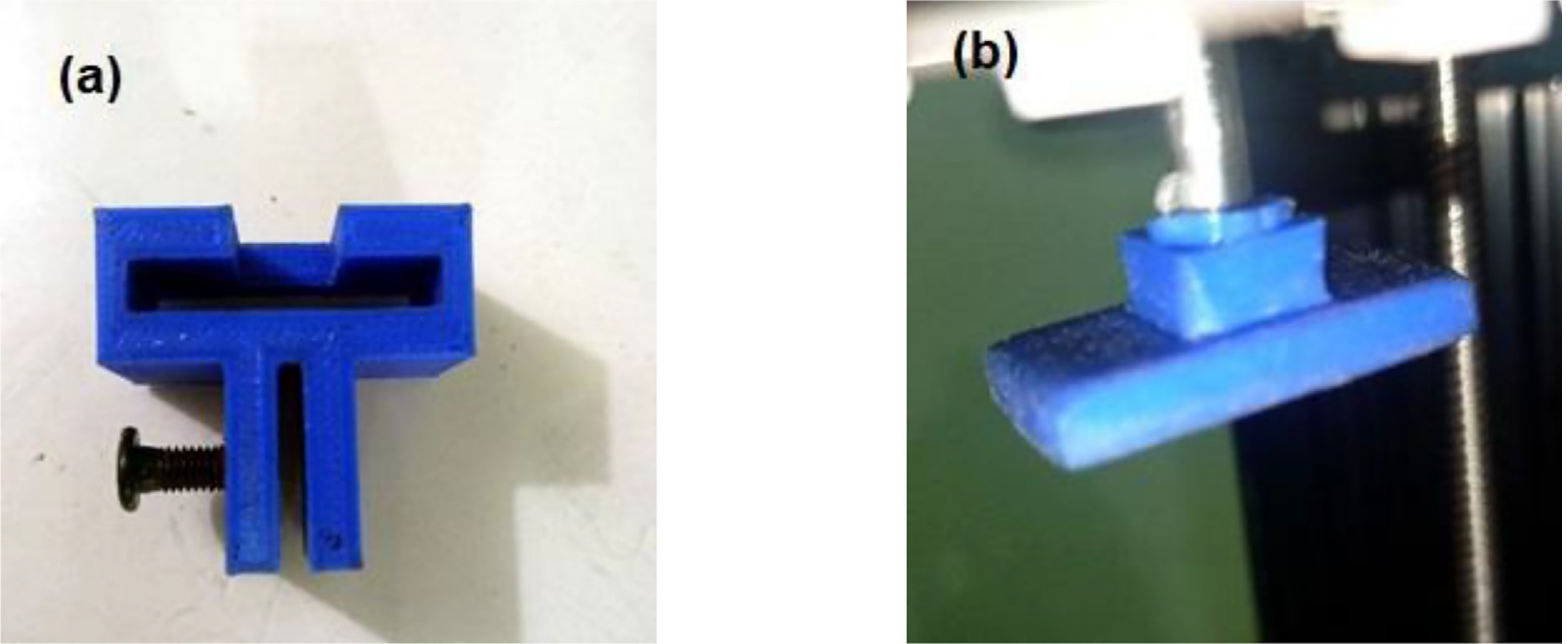
a) 3D printed stepper holder b) 3D printed slide holder.

Electronic module

• Power supply

**Fig. 15:**
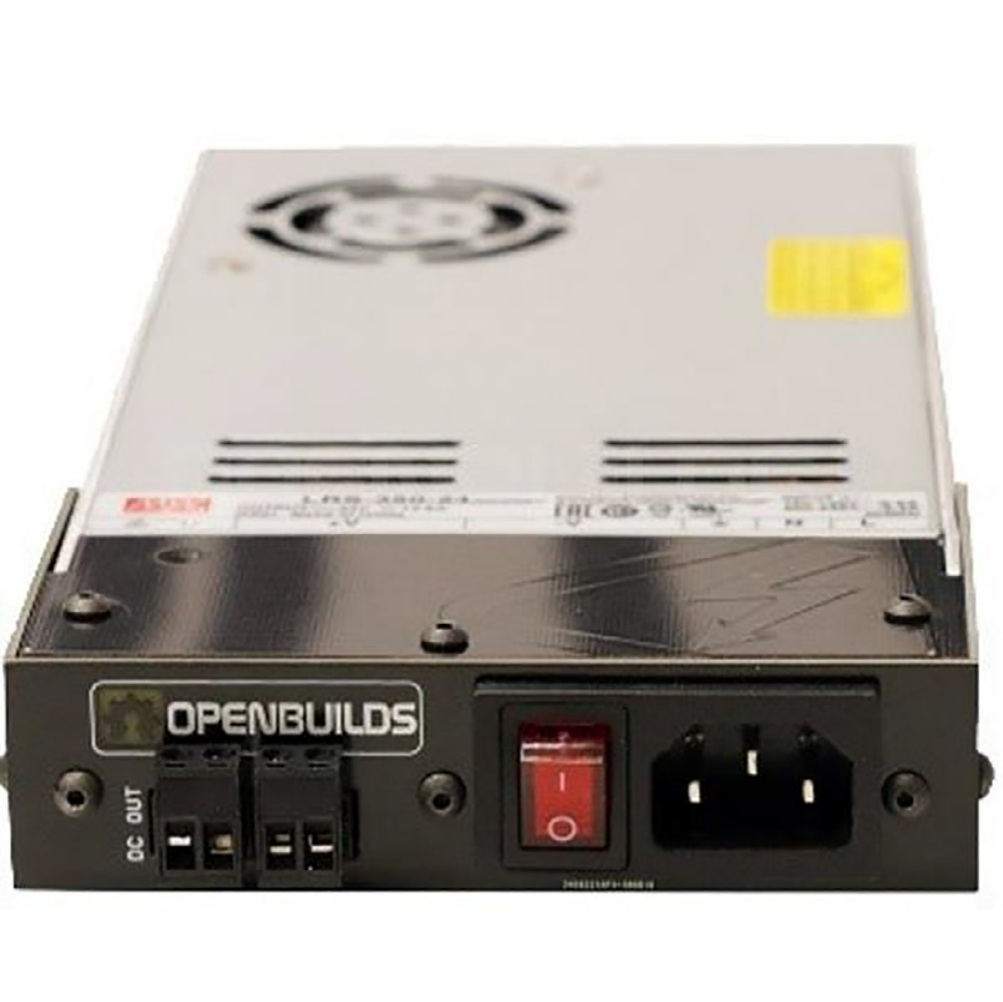
24 V Meanwell Power Supply Bundle (Courtesy: OPENBUILDS).

The power supply module used in the system is shown in Fig. 15.

▪ PCB and control unit
1. Print the PCB (Printed Circuit Board) using a double-sided FR4 board from any PCB printing service provider.
2. First, solder the SMD components provided in the part list.
3. After that, solder THT (through hole) components.
4. Then solder the connectors for the output ports.
5. Adjust the output voltage of LM2596 to 5 V using the potentiometer in the module before placing the MCU and A4988 Stepper Driver.
6. Solder the I2C module to the LCD module.
7. Adjust the contrast of the LCD using the potentiometer of the I2C module and set the address of the I2C module according to the chip in programming.

**Fig. 16:**
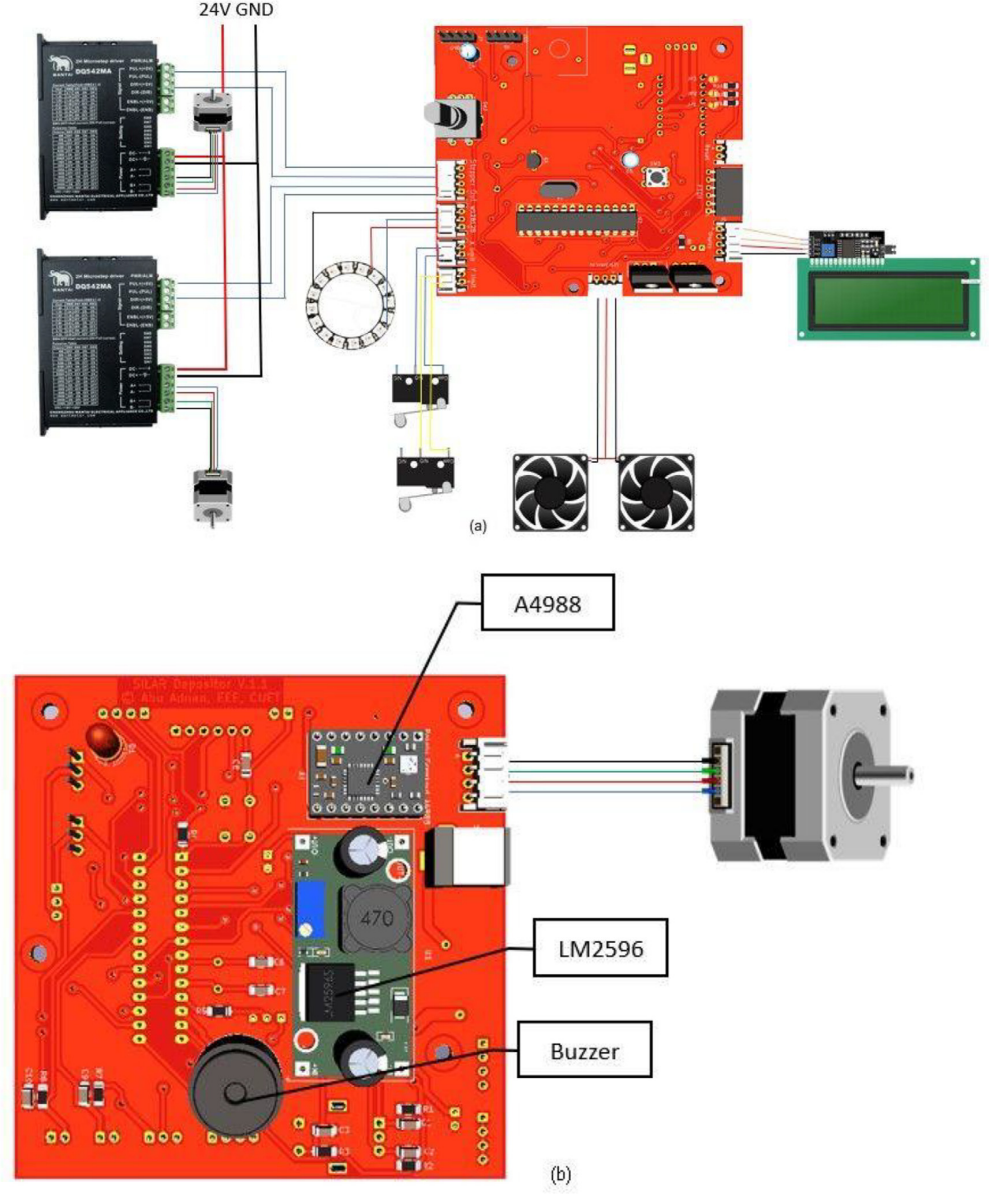
a) Electrical system architecture (front) b) Electrical system architecture (back).

The overall electrical architecture is shown in Fig. 16.

## Operation instructions

### Initial setup

Once the mechanical structure and electrical assembly are prepared, we need to upload the operation code into the system through the upload port of the control panel. The given code files in the “Design Files” section need to be uploaded using the Arduino IDE on a compatible computer. Before uploading, we need to select the correct port number from the IDE. The uploading process generally takes about 20 s to complete. Before uploading, we need to calculate the X-axis length and Y-axis length in terms of the number of steps of the stepper motor. For this, a single run along the X-axis and Y-axis is performed and the number of steps of the stepper motor is calculated. For our device, we used 3200 micro steps setting in the stepper driver and found the X-axis length to be 42,860 steps, whereas the Y-axis length is found to be 150,000 steps. These step values need to be updated in the code file before uploading.

### Device startup and operation

**Fig. 17:**
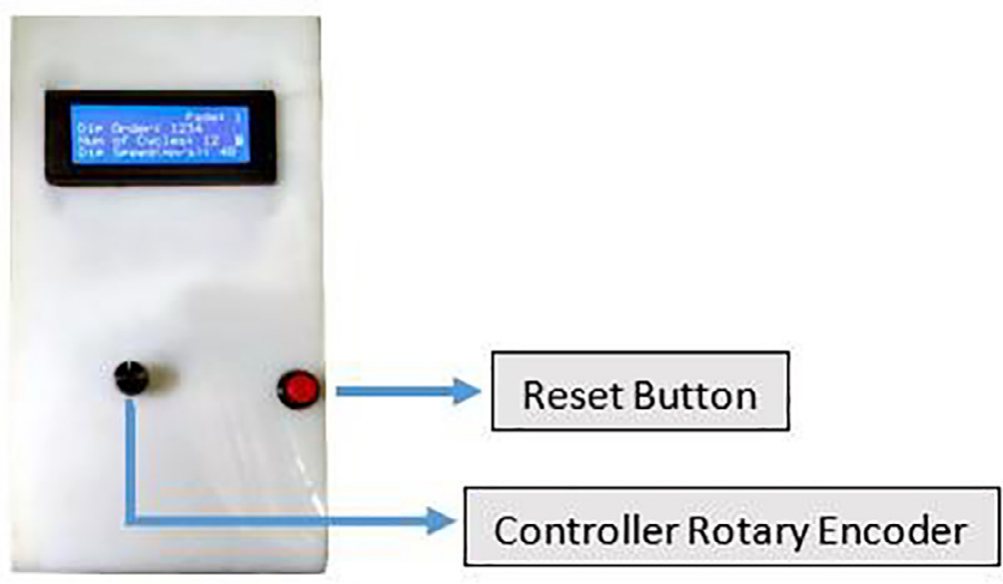
Control panel overview.

To start the device, simply turn on the power button on the back of the control panel. The whole machine is controlled by one reset button and one controller rotary encoder on the control panel (Fig. 17).

**Fig. 18:**
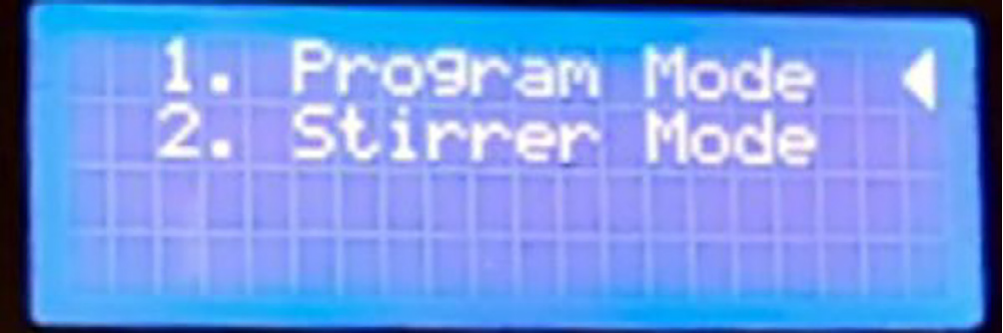
Operation modes.

To ensure continuous operation, the device needs to run on an uninterrupted power backup (UPS). There are two modes of operation available for the device to operate as shown in Fig. 18: i) Program Mode and ii) Stirrer Mode. On starting the device, the control panel presents these two options for the user to choose.

The selection pointer jumps between the two options when the knob of the rotary encoder is rotated clockwise and counter-clockwise. Any of the two modes can be selected by pressing the button on the rotary encoder.

### Program mode

**Fig. 19:**
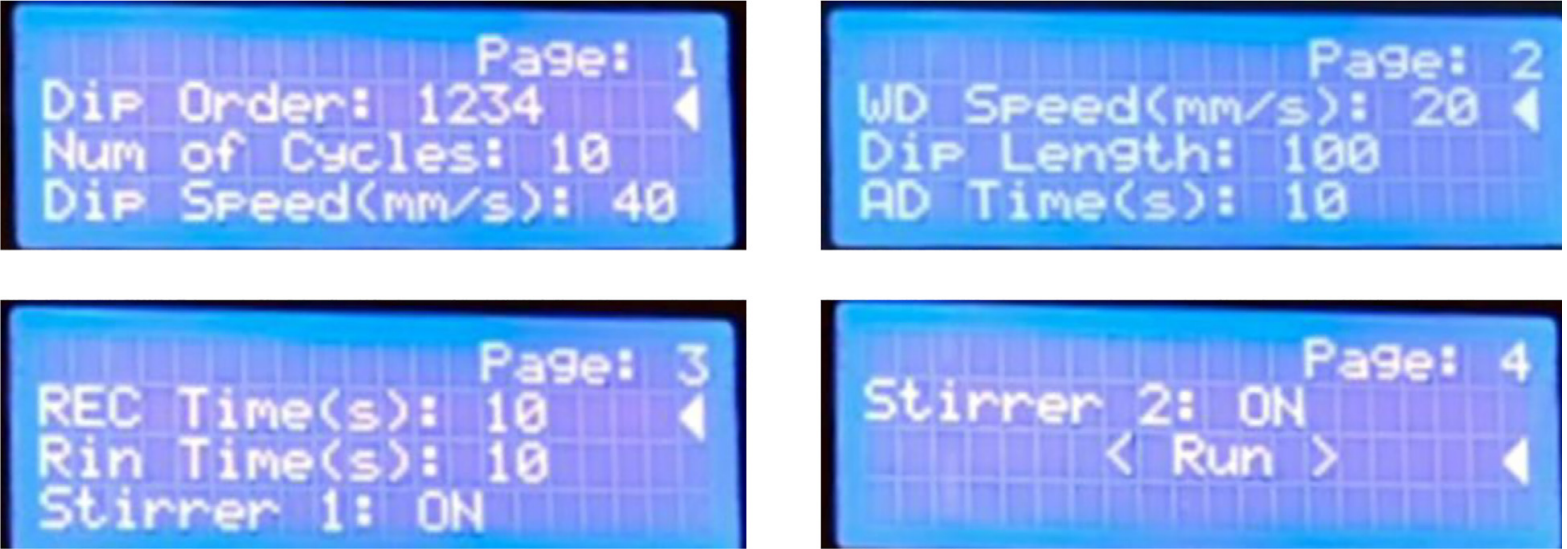
Setting parameters of SnabFib.
C. To modify a certain setting the selection cursor needs to be on that line of setting and to modify the value the rotary encoder switch needs to be pressed to enter the editing mode (Fig. 20).

The device can be run in program mode in the following way:

A. Inside the program mode, there are several settings a user can tweak to suit the necessities of the operation. The settings are described below:

Program mode settings:

1. **Dip Order:** Consists of 4 digits specifying the sequence in which the dipping of the substrate will take place. The digits can be repeated more than once. Thus, different combinations of the digits define different dip orders that the SILAR cycle needs to be operated on.
2. **Num of Cycles:** Specifies the number of times a SILAR cycle will be repeated. The value ranges from 1 to 999. By varying the number of cycles, the thickness of the film to be deposited can be changed.
3. **Dip Speed:** Specifies the speed in mm/s at which the substrate will be dipped inside the cationic beaker, the anionic beaker, and the rinsing beakers. This speed value ranges from 1 to 40 mm/s.
4. **WD Speed:** Specifies the withdrawal or removal speed of the substrate in mm/s from the beakers. To ensure the substrate is withdrawn from the beakers in a safe and vibration-free way, the value of this speed needs to be tweaked as necessary. This speed value ranges from 1 to 40 mm/s.
5. **Dip Length:** Specifies the length up to which the substrate will be dipped inside the beakers. This setting varies with the dimension of the slides used at the time of deposition. And the value of this setting ranges from 1 to 999 mm.
6. **AD Time:** Sets the adsorption time, i.e., the duration of time in seconds the substrate will be dipped inside the cationic solution. The value ranges from 1 to 100 s.
7. **REC Time:** Sets the reaction time, i.e., the duration of time in seconds the substrate will be dipped inside the anionic solution. The value ranges from 1 to 100 s.
8. **Rin Time:** Sets the rinsing time, i.e., the duration of time in seconds that the substrate will be rinsing in the deionized water beaker.
9. **Stirrer 1:** ON/OFF setting specifying if the stirrer 1 will be ON or OFF during the operation. If it is set to ON, the stirrer will be automatically turned off each time the substrate is dipped inside the beaker at position 1 and turned on after the substrate is out of the beaker.
10. **Stirrer 2:** ON/OFF setting, specifying if the stirrer 2 will be ON or OFF during the operation. If it is set to ON, the stirrer will be automatically turned off each time the substrate is dipped inside the beaker at position 3 and turned on after the substrate is out of the beaker.
B. The mentioned setting parameters are spread through different pages in the user interface as shown in Fig. 19. To select a particular setting from a specific page the knob of the rotary encoder needs to be rotated clockwise or counter-clockwise to go back and forth in the settings list.

**Fig. 20:**
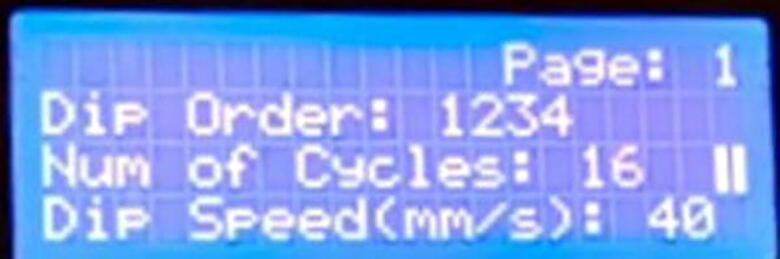
Editing mode in the user interface.

In the above figure the parameter “Num of Cycles” is in the editing mode. Then the value can be changed by the rotation of the knob of the controller rotary encoder. The encoder switch needs to be pressed again to exit and save the new setting value. Once changed the system stores all values in the system memory so that every time the device starts with previously set settings values.

**Fig. 21:**
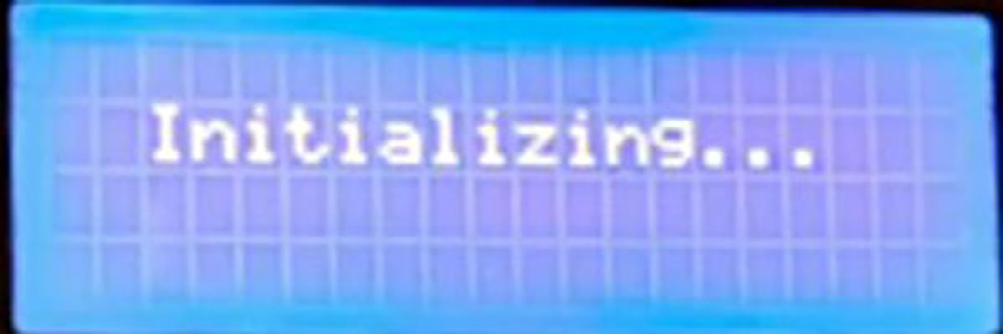
Initialization screen.

After saving the above settings choosing the last option “Run” from page 4, starts the SILAR operation. At first, the device starts initialization of the x-axis and y-axis and the user interface notifies the user about this initialization stage as shown in Fig. 21.

**Fig. 22:**
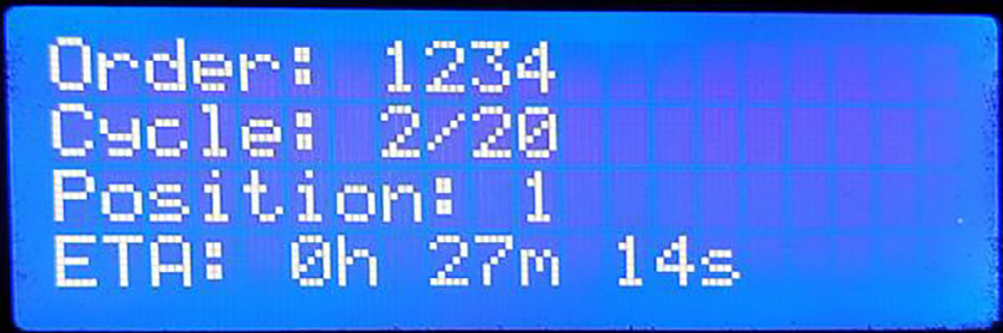
Running status screen.
E. After completion of the operation, the system returns to the initial page to select the mode of operation for the next deposition. The deposition process can be stopped before the completion of the total number of cycles, by pressing the reset button.

D. After initialization during the operation, the status screen shown in Fig. 22 notifies the user about the current cycle number, the current position of the substrate head, and the remaining time needed to complete the operation.

### Stirrer mode

**Fig. 23:**
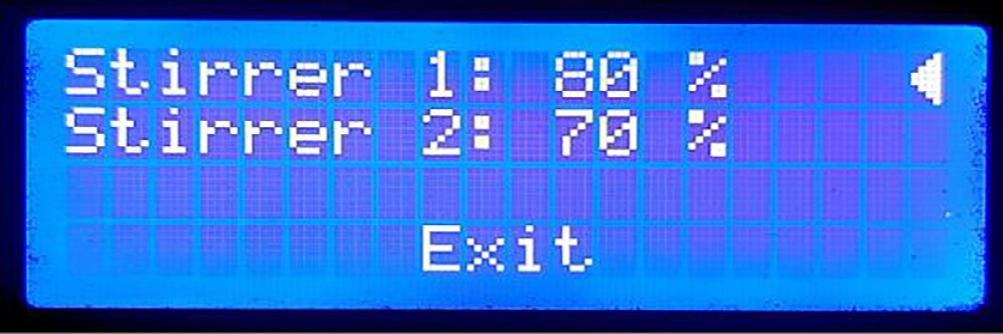
Stirrer mode screen.

In the stirrer mode, the magnetic stirrer can be operated without running the whole system as. This mode is useful for cases when only chemical stirring is required. The following two settings are available in this mode of operation as shown in Fig. 23.

i) **Stirrer 1:** Specifies the speed of stirrer 1 in the percentage of the maximum speed. The value ranges from 0 to 100 %. When the value is set to 0 % the stirrer turns off.
ii) **Stirrer 2:** Specifies the speed of stirrer 2 in the percentage of the maximum speed. The value ranges from 0 to 100 %. When the value is set to 0 % the stirrer turns off.

The reset button needs to be pressed once to exit from the “Stirrer Mode”.

## Validation and characterization

After completion of our construction, we validated the performance by depositing a zinc-sulfide (ZnS) thin film on a soda lime glass substrate and characterized it.

We had been supplied with a properly degreased and ultrasonically cleaned corning soda lime glass substrate, as outlined in [12], having dimensions of 75 mm × 25 mm. Standard cleaning procedures were maintained using organic solvents, dried by N_2_ jet flow, and annealed on the hotplate at 120 °C. This glass substrate was then clipped with a substrate holder. Four 150 mL glass beakers were taken. Two beakers were poured with deionized water for rinsing purposes and set at the 2nd and 4th positions (from the left of the operator) upon the beaker holder base. A cationic solution was prepared by dissolving ZnSO_4_ salt in 150 mL of deionized water with a concentration of 0.3 M for adsorption of cation Zn^2+^ ions and setting the beaker containing this solution at 1st position. The anionic solution was prepared by dissolving Na_2_S in 150 mL of deionized water with a concentration of 0.05 M. The reaction beaker containing the anionic solution was then set at the 3rd position.

The control parameters were set as shown in Table 1:

**Table 1:** Parameter Settings of SILAR depositor for a given recipe.

Parameter Name	Parameter Value
Dip Sequence	1234
Number of Cycles	50
Dip Speed	40 mm/s
Reaction Time	10 sec
Rinse Time	10 sec
Stirrer 1	On
Stirrer 2	On

After completion of the SILAR deposition cycles, we unmounted the glass substrate, dried by N_2_ jet, and this as-deposited thin film was annealed on a hot plate at 300 °C for an hour. Then samples were cut by a diamond cutter to the proper sizes as required for characterization.

The X-ray diffraction patterns of the films were recorded using a PANalytical-X’pert PRO instrument, a diffractometer in the scanning range 20°-80° using Cukα radiations with wavelength 1.5406 Å. SUPRA 5S-CARL Zeiss, Field Emission Scanning Electron Microscope (FESEM) with an integrated Energy Dispersive X-ray (EDX) spectroscopy was used for the determination of morphology and chemical composition. Dektak XT profilometer was used to determine the film thickness.

The thickness of the annealed ZnS thin-film was found to be around 300 nm.

**Fig. 24:**
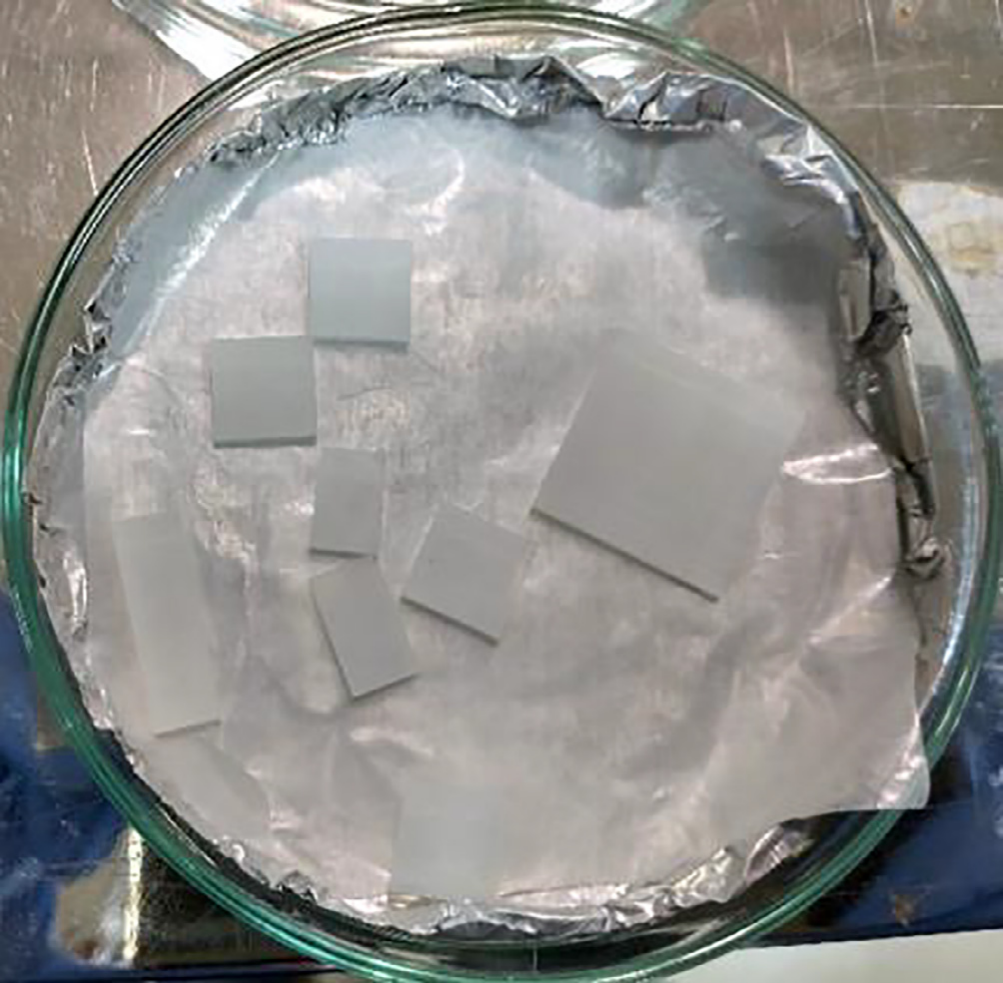
Preparing deposited films for characterizations.

The deposited ZnS film by SILAR method using SnapFib is shown in Fig. 24.

**Fig. 25:**
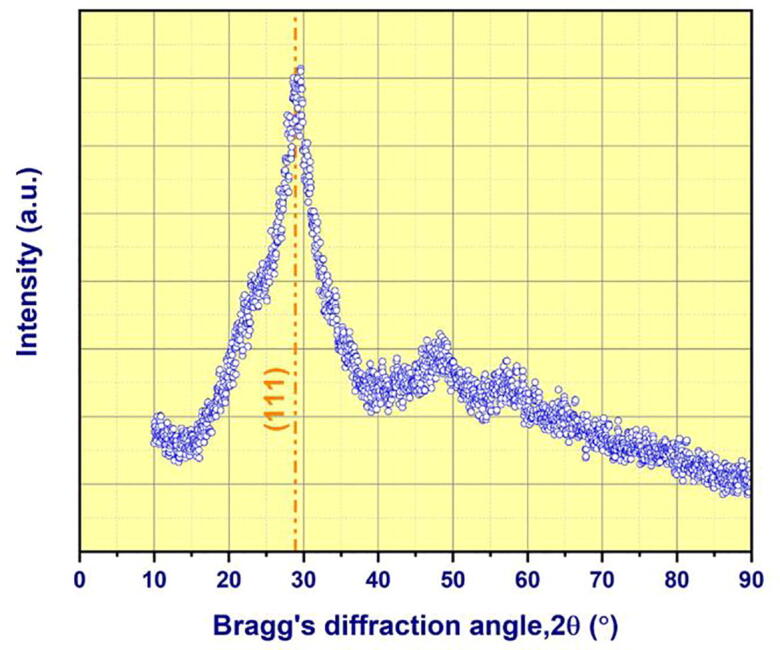
XRD pattern of ZnS thin film deposited by SILAR method using SnapFib.

Fig. 25 shows Bragg’s diffraction peaks of ZnS thin-film as obtained from XRD characterization deposited by the SILAR method. The film exhibits a dominant peak at $2\theta \;=28.92^{{}^{\circ}}$ which corresponds to cubic phase β-ZnS [13] oriented along the (1 1 1) plane. The other β-ZnS was observed at $2\theta =47.93^{{}^{\circ}}$ with poor peak intensity. We used the Lorentz peak fitting function to obtain crystallographic data from XRD results for the dominant peak at $2\theta =28.92^{{}^{\circ}}.$ The following table compares our results with other reported research which used the SILAR method for ZnS thin-film deposition.

An overall comparison between the results found from this experiment and those of other similar works is shown in Table 2.

**Table 2:** Comparison between our work and several other similar works.

Reference	Method	Plane	2θ (°)	d(Å)	FWHM **(**°)	D (nm)	a = b = c (Å)	$\varepsilon \left(\times 10^{-3}\right)$	$\delta (\times 10^{11}cm^{-2})$
[14]	SILAR	111	29.24	3.19	0.63	12.4	5.52	10.53	5.89
[13]	SILAR	111	28.60	3.14	–	–	5.44	–	–
[15]	SILAR	111	29.00	3.12	–	–	5.41	–	–
[16]	SILAR	111	28.58	3.12	–	–	5.41	–	–
Our work	SILAR	111	28.92	3.09	6.978	1.18	5.34	118.07	723.60

2θ= Bragg’s diffraction angle.

d = Inter-planner distance.

FWHM = Full-width at Half Maximum.

D = Crystallite size.

D is obtained by the well-known Debye-Scherrer formula $D=\frac{k\lambda }{FWHM\times cos\theta }$ [17].

wherek=0.9

λ=0.154046nm

θinradians

$$FWHM\;\left(in\;\;radian\right)$$

Lattice constants = a, b, c.

Microstrain, ɛ$=\frac{FWHM}{4tan\theta }$

Dislocation density, δ $=\frac{n}{D^{2}}$; n = 1.

**Fig. 26:**
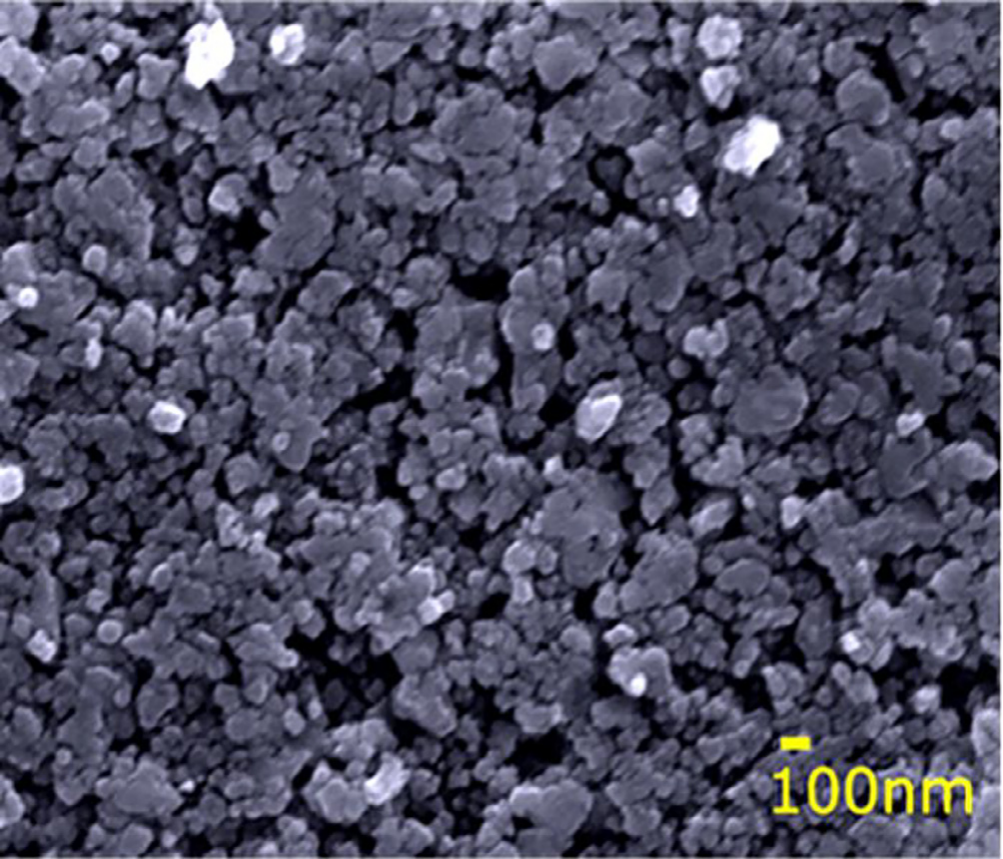
FESEM image of ZnS thin film deposited by SILAR method using SnapFib.

Fig. 26 shows the FESEM micrographs of our SILAR method deposited thin film. From the FESEM we have been able to visualize different topographic characteristics and the goodness of the region in the film surface. This FESEM approach is very common for researchers to determine structures of size smaller up to even 1 nm. [18].

**Fig. 27:**
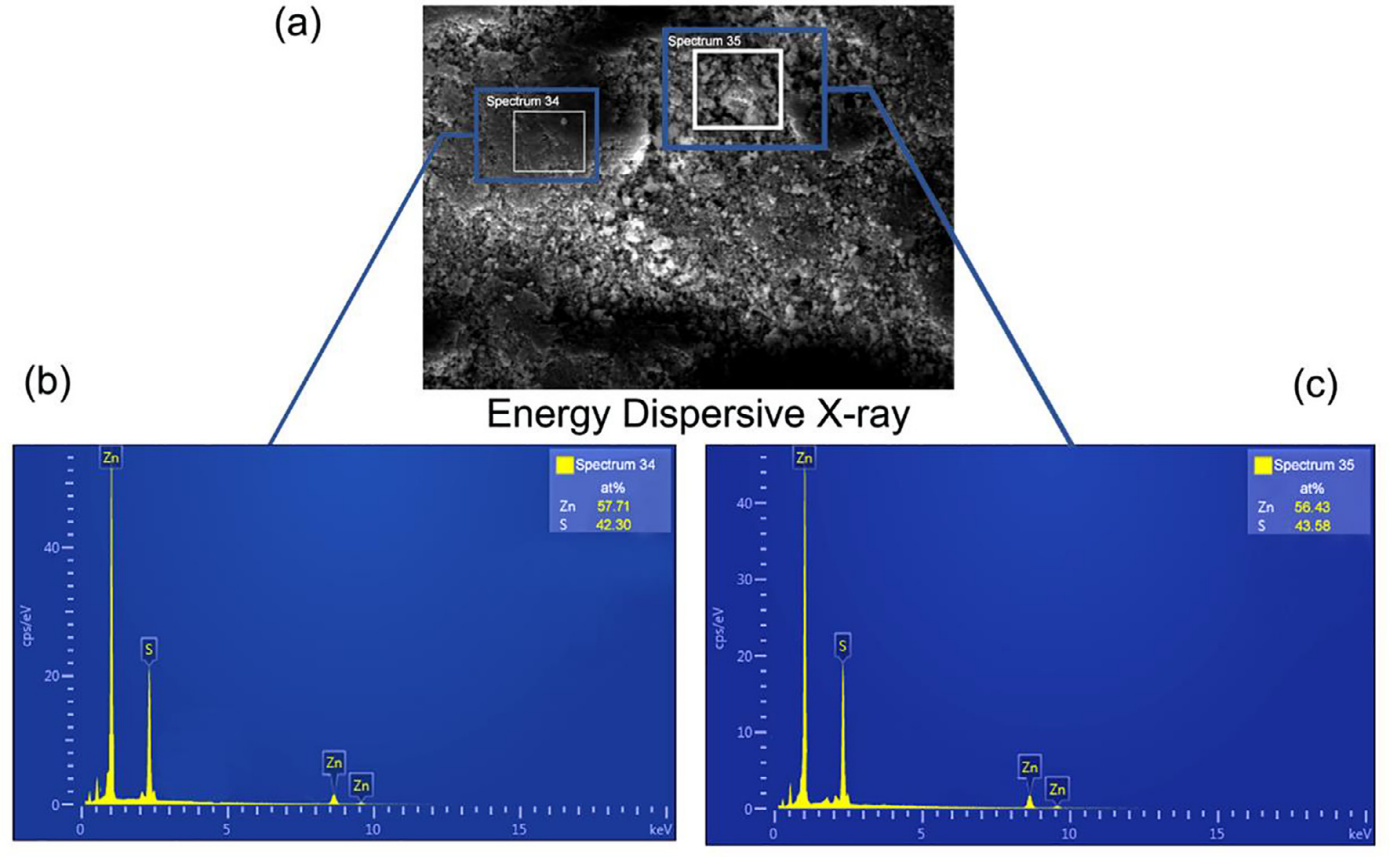
Typical EDX spectrum for deposited ZnS thin film a) selected spectrum sites b) spectrum 34c) spectrum 35.

Fig. 27 shows the EDX (Energy Dispersive X-ray) spectroscopy plots for the ZnS films. EDX or Energy dispersive X-ray spectroscopy uses transitions of electrons to characterize materials. Since each material has its own atomic structure, in the EDX there will be a different collection of peaks on the electromagnetic emission spectrum. From the EDX data we observe two sharp peaks respectively for Zn and S. So, this tells us that the deposited film is of a binary compound.

Table 3 shows the percentage of Zn and S in EDX spectroscopic data for deposited ZnS thin-film. The results are in good agreement with the references, which used a similar approach. The precision of the results depends not only on the quality of the chemical solutions but also on the fact that the designed system has very smooth motion and is free of vibrations.

**Table 3:** The atomic percentage of Zn and S atoms in the deposited film from the EDX data.

Spectrum	Zn (at %)	S (at %)
spectrum-34	57.71	42.30
spectrum-35	56.43	43.58

## Ethics statements

This work doesn’t involve the use of human and animal subjects.

## CRediT authorship contribution statement

**Ashoke Kumar Sen Gupta:** Conceptualization, Methodology, Original draft preparation. **Abu Adnan:** Hardware, Editing. **Shantanu Bhattacharjee:** Software, Editing. **Nipu Kumar Das:** Validation. **M.A. Matin:** Supervision, Validation. **Muhammad Quamruzzaman:** Supervision, Writing – review & editing.

## Declaration of Competing Interest

The authors declare that they have no known competing financial interests or personal relationships that could have appeared to influence the work reported in this paper.
